# The importance of social environment in preventing smoking: an analysis of the Dead Cool intervention

**DOI:** 10.1186/s12889-019-7485-7

**Published:** 2019-08-28

**Authors:** Jennifer Badham, Helen McAneney, Laura Dunne, Frank Kee, Allen Thurston, Ruth F Hunter

**Affiliations:** 1 Centre for Public Health, Queen’s University Belfast, Institute of Clinical Sciences, Block B, Royal Victoria Hospital, Belfast, BT12 6BA UK; 2 Centre for Evidence and Social Innovation, Queen’s University Belfast, University Road, Belfast, BT7 1NN UK; 3 School of Social Sciences, Education & Social Work, Queen’s University Belfast, College Green, Belfast, BT7 1LN UK

**Keywords:** Social contagion, Smoking prevention, Adolescents

## Abstract

**Background:**

An adolescent’s perceptions of their family’s and friends’ smoking attitudes and behaviour can influence their own uptake of smoking. There are two broad sources of such social influence: observing the behaviour directly, and assimilating attitudes.

**Methods:**

We analysed data collected for the evaluation of Dead Cool, a school based smoking prevention intervention in Northern Ireland (*n*=480 in 20 clusters). The main analysis fits three nested logistic regression models predicting pre-intervention susceptibility to taking up smoking, as reflected in responses to three attitudinal questions. Model 1 includes only personal characteristics as explanatory factors. Model 2 adds the behaviour of friends and family that would provide an opportunity for social influence through observational learning. Model 3 adds the susceptibility of friends.

**Results:**

Each additional group of variables improved the model fit (with reduced AIC and BIC). However, in the final model, only three variables were found to be statistically significant (*p*<0.05) in predicting susceptibility to smoking initiation: rebelliousness (OR [1.1,1.3]) from the personal characteristics group; and, in the observational learning group, being friends with a smoker (OR [1.0,2.9]) and frequency of being in the same room or car with someone smoking (OR [2.0,9.0] for most frequent). Adding the two measures of diffusion of susceptibility through the friendship network improved the model fit, but neither was found to be statistically significant.

**Conclusions:**

The analysis provides additional evidence to support policies that could reduce children’s exposure to smoking behaviour, and potential subsequent smoking initiation. No conclusions could be drawn about the diffusion of smoking attitudes through the school friendship networks of children.

**Electronic supplementary material:**

The online version of this article (10.1186/s12889-019-7485-7) contains supplementary material, which is available to authorized users.

## Background

It has long been established that an adolescent’s perceptions of their family’s and friends’ smoking attitudes and behaviour influence their chances of becoming smokers themselves [1–3]. Such influence operates directly on smoking behaviour, but also indirectly through intention to smoke [4, 5]. Potential mechanisms for this influence include exposure to behaviour modelling and smoking tolerant social norms (from Social Cognitive Theory, [6]).

Peer influence is only one of many factors considered in the diverse theoretical lenses that have been applied to adolescent smoking [4]. Some of these theories emphasise individual characteristics such as gender or response to stress, while others highlight the relationship between an adolescent and social groups in the form of social bonding or support rather than peer influence. Of course, these factors may also contribute to adolescent smoking behaviour in combination [4].

Several studies have used social network analysis methods to investigate the relationship between adolescent smoking, their network of friends, and the smoking status of those friends [7] (Montgomery S, Donnelly M, Bhatnagar P, Carlin A, Kee F. Hunter RF: Peer social network processes and adolescent health behaviours: a systematic review, submitted). A recent meta-analysis [8] identified 39 studies that estimated the effect of the smoking status of nominated friends (referred to as close friends in the analysis) on smoking initiation or continuation. Additionally, there is strong evidence that smoking and friendship co-evolve during adolescence [9]; non-smokers with friends who smoke are more likely to become smokers, and smoking status is one of the factors contributing to the choice of friends. In network theories, similarity within friendship groups is referred to as homophily, and these mechanisms are referred to as influence and selection effects respectively [4, 10].

However, these analyses consider smoking behaviour, or attitudes formed after smoking behaviour has been initiated. To the extent that attitude contributes to behaviour, such as posited by the well-established Theory of Planned Behaviour [11], peer influence studies that examine smoking cannot detect network diffusion of attitudes that predispose such behaviour. In particular, if similar attitudes to smoking contribute to the selection of friends and those friendship networks predate smoking behaviour, then the diffusion of smoking behaviour will appear as an influence effect.

Network studies have also found effects that are unrelated to the smoking status of friends. For example, smoking is positively associated with popularity (the number of friend nominations received), but also with having no friends [7].

In Northern Ireland in 2013, 6% of children aged 11-16 smoked at least monthly, and 12% believed it was okay for someone their own age to smoke at least weekly [12]. Of those who have ever had a cigarette, 77% had their first cigarette aged between 12 and 15 years, spread approximately equally for each age [13].

Dead Cool is a school based smoking prevention programme developed by Cancer Focus Northern Ireland [14]. It comprises materials for four lessons to be delivered by teachers in their own classes. Teacher training is also provided. The lessons explicitly cover social and environmental influences on smoking, including smoking behaviour of friends and family, and media placement of cigarettes. It is intended to encourage students to recognise and challenge such influences.

In this study, we used data from the evaluation of Dead Cool to investigate the role of the social environment in attitude formation in young adolescents prior to initiation in smoking behaviour. We examine the association between smoking susceptibility of young non-smokers (defined below as a composite of smoking related attitudes, see [15]) and two types of social environment influences. Observational learning measures describe the (perceived) smoking behaviour of friends and family. Diffusion effects measure the potential diffusion of susceptibility over a friendship network.

## Methods

Dead Cool was evaluated using a randomised controlled trial in 20 school classes (of size 16 to 31) with a total of 480 Year 9 students (aged 12 or 13 years). The classes were recruited from 17 schools. For 14 schools able to be paired by school characteristics, one class was selected from each school and assigned randomly to the intervention or control group. Two classes were selected from the other three schools as a control and intervention pair, because a second school with similar characteristics was not available. Full details are available in the evaluation [14].

A 28 question survey was completed by students in the trial pre/post intervention and at three months follow-up. This questionnaire covered demographic information, own smoking behaviour, smoking by friends and family, and attitudes to smoking (see Additional file 1). Carbon monoxide in exhaled breath was also tested to confirm smoking status. One question asked students to nominate their five closest friends in their class.

All data processing and statistical analyses were performed in R ([16], [version 3.2.2]).

### Outcome measure: susceptibility to smoking

As expected in this age group, smoking prevalence was very low in the study population, consistent with the smoking prevention objective of the intervention. Only 19 students at pre-intervention reported that they smoked, and seven students recorded a carbon monoxide level in exhaled breath that indicated smoking. The main outcome variable for this study is therefore whether the student is susceptible to smoking (yes or no). Susceptible non-smokers have been shown to have approximately twice the likelihood of smoking uptake as those not susceptible [15].

Susceptibility is constructed from three questions:

- Do you think you will try a cigarette soon?
- If one of your best friends were to offer you a cigarette, would you smoke it?
- Do you think you will smoke a cigarette at any time in the next year?

The student was coded as not susceptible if they answered ‘No’ (from three choices), ‘Definitely not’ (from five choices) and ‘Definitely not’ (from five choices) respectively to these questions. The student was coded as susceptible with any other set of responses. If the student failed to respond to one or more of these three questions, susceptibility was coded as missing.

### Explanatory measures

Ten explanatory measures were examined in three groups: personal characteristics, social environment, and diffusion of susceptibility. Both the social environment and diffusion groups concern social influence, however they differ in potential mechanisms for that influence. The social environment group comprises measures of observable behaviour, whereas susceptibility cannot be perceived by other students.

The personal characteristics variables comprised gender, relative deprivation, rebelliousness and life satisfaction, which are known to be associated with smoking [3, 17]. Deprivation is the Northern Ireland Multiple Deprivation Measure [18] decile, coded from residential address. Rebelliousness was scored from 0-12 by summing four items (see question 23) asking how well statements such as ‘I get in trouble at school’ describe themselves. Life satisfaction was scored from 0-20 by summing agreement with five items such as ‘My life is going well’ (see question 22).

The observational learning variables comprised four measures of behaviour associated with smoking uptake [3, 5], whether anyone in their immediate friends and family is a smoker, and the frequency of exposure to smoking in the same room or car. Two measures aggregated family members and friends respectively from the detailed list of people they know who smoke (see question 12) to construct binary responses to whether at least one family member smokes (mother, father, step-parent, brother or sister) and whether at least one friend smokes (boyfriend or girlfriend, some friends of my own age, some older friends, some younger friends). The list of people they know who smoke was separately combined with the list of who the student lives with (see question 5) to construct a binary measure of whether the student lives with at least one smoker.

Four questions (see 14 to 17) asked about how often the student was in the same room or car as someone smoking. The responses were combined into a three level measure of smoking exposure: ‘Frequently’ if at least one was in the two most frequent categories, ‘Sometimes’ if none were in the frequent categories and at least one was in the middle two categories, and ‘Never’ if the response to all four potential situations was ‘Never in the past year’.

The diffusion of susceptibility variables combined network data collected in the study with smoking attitude responses of the nominated network members, to permit investigation of potential diffusion of attitudes over personal networks. Each participant was asked to nominate up to five friends from their class. The nominees were coded using each student’s unique identifier, with a separate code where a nominee was unable to be matched to a trial participant. The coded nominations were used to create directed edges in a social network of participants (using the igraph package in R). For each student, the measure ‘out-susceptible’ was calculated as the proportion of their nominees who were susceptible. Separately, the measure ‘in-susceptible’ was calculated as the susceptible proportion of students who nominated that student.

Note that there is no expectation that the nominated friends are the same as the friends referred to in the questions about the smoking status of friends, there may not even be overlap. The nominations are restricted to people in the same school class. The questions about the smoking status of friends refer to whoever the respondent perceives as a friend.

### Statistical analysis - pre-intervention smoking susceptibility

Preliminary analyses constructed simple logistic regression models of susceptibility for each of the ten explanatory factors of interest individually. Those factors that attained a statistical significance level of *p*<0.1 were retained for further modelling.

Multi-variate mixed-effects logistic regression models (glmer procedure in the lme4 package) were constructed in three stages. For all models, school class was included as a random effect, as students were randomised at the class level. These models explored the association between pre-intervention susceptibility with the retained explanatory factors in groups, starting with the personal characteristics, then adding the observational learning and diffusion of susceptibility variables at later stages.

Model 1 included all personal characteristics retained from the preliminary analysis. Model 2 included the statistically significant (*p*<0.05) variables from Model 1 and added retained observational learning measures. Model 3 included the statistically significant (*p*<0.05) variables from Model 2 and added the retained diffusion of susceptibility variables.

The three multivariate models were compared using their Akaike information criterion (AIC) and Bayesian information criterion (BIC; both obtained using the glmerMod procedure in the lmerTest package) to assess whether the additional explanatory variables improved the model fit.

### Statistical analysis - change in smoking susceptibility

We examined change in susceptibility over the three waves with two approaches. The initial analysis used stochastic actor-oriented models, the standard method to estimate the relationship between network structure and behaviour of individuals within the network [19, 20]. We applied these models to understand the diffusion of susceptibility over the three time points. The standard approach is to construct separate models to estimate effects within each network, and then conduct a meta-analysis to estimate effects that differ between networks.

We constructed a separate model (with the RSiena package version 1.1 [21]) for each class where at least 80% of the students provided identifiable friendship nominations for all three time points. Susceptibility was included in the model as the behaviour variable to be explained, with susceptibility of friends as the key explanatory behaviour co-variate. Gender and rebelliousness scores were included as explanatory factors for network structure (that is, friendship formation) as well as typical network structure effects such as transitivity. In addition, intervention group was retained for the meta-analysis to allow the network diffusion of susceptibility to differ between the intervention and control groups.

Convergence of these stochastic actor-oriented models was poor. Therefore, we also constructed mixed-effects logistic regression models (glmer procedure in the lme4 package) to investigate susceptibility at post-intervention or follow-up adjusting for prior susceptibility. Other explanatory variables were intervention group (to adjust for any effect of the Dead Cool intervention), the statistically significant factors from the susceptibility at pre-intervention model, and the network influence variables. These models are simpler than stochastic actor-oriented models, ignoring network changes between survey waves. As for the baseline susceptibility, school class was included as a random effect.

## Results

### Descriptive analysis

At pre-intervention baseline, 141 of 480 students (29%) were assessed as susceptible to smoking, 290 (60%) as not susceptible, with the remaining 49 (10%) unable to be assessed. The susceptible students were more likely to have smokers in their social circle and be in the presence of smoking more frequently than non-susceptible students (see Table 1).

**Table 1:** Characteristics of students by susceptibility to smoking, at pre-intervention

	Population	Susceptibility^1^	
		Yes	No
Students	480	141	290
Personal characteristics			
Gender (M/F) ^2^	251/228	75/66	144/145
Deprivation decile: mean [SD]	0.41	0.43 [0.31]	0.40 [0.29]
Rebelliousness: mean [SD]	3.9	4.9 [2.6]	3.4 [2.5]
Life Satisfaction: mean [SD]	14.5	14.2 [2.4]	14.6 [2.3]
Nominations received: mean [SD]	3.6	3.7 [2.3]	3.8 [2.4]
Observational learning			
Live with a smoker	43%	54%	37%
Smoker in family	48%	60%	42%
Friends with a smoker	34%	52%	26%
Smoking presence: Never	26%	11%	34%
Smoking presence: Sometimes	29%	27%	30%
Smoking presence: Frequently	45%	62%	37%
Diffusion of susceptibility			
Nominated friends susceptible	34%	41%	29%
Nominators susceptible	34%	45%	29%

^1^Susceptibility could not be assessed at pre-intervention for 49 students as they were either absent on the survey date or did not respond to at least one of the three relevant questions

^2^One student did not identify their gender

Overall, there was a small increase over time in the number of students assessed as susceptible to smoking, but there was change in both directions. Between pre-intervention and post-intervention, 44 students became susceptible and 34 became not susceptible. Between post-intervention and follow-up, 39 became susceptible and 31 became not susceptible (see Table 3).

### Susceptibility at pre-intervention

Univariate regressions found no evidence that Gender (*p*=0.67) or Deprivation (*p*=0.22) explain pre-intervention susceptibility in the Dead Cool study and they were excluded from further analysis. The remaining eight variables were used to construct three multivariate regression models (results at Table 2).

**Table 2:** Odds ratios for susceptibility to smoking by personal and social environment characteristics (with 95% confidence interval)

	Model 1	Model 2	Model 3
Personal characteristics			
Rebelliousness	1.2 (1.1-1.4)	1.2 (1.1-1.3)	1.2 (1.1-1.3)
Life Satisfaction	0.9 (0.9-1.0)		
Observational learning			
Live with a smoker		1.9 (0.7-5.7)	
Smoker in family		0.5 (0.2-1.4)	
Friends with a smoker		2.0 (1.2-3.3)	1.7 (1.0-2.9)
Smoking presence: Never		Reference	Reference
Smoking presence: Sometimes		2.4 (1.2-5.3)	3.0 (1.4-7.4)
Smoking presence: Frequently		4.2 (2.0-9.2)	4.2 (2.0-9.9)
Diffusion of susceptibility ^1^			
Nominated friends susceptible			2.9 (1.0-8.8)
Nominators susceptible			1.0 (0.3-3.1)
Model fit: AIC	456	429	383
Model fit: BIC	472	460	413

^1^These variables are proportions; a unit change is the difference between none and all nominations or nominators being susceptible

Model 1 included the personal characteristics explanatory variables, of which only Rebelliousness and Life Satisfaction remained. Only Rebelliousness was significant (*p*<0.001) and it was combined with the observational learning variables for Model 2.

Model 2 was a better fit with the dataset than model 1 (AIC improved from 456 to 429), indicating the importance of the observational learning factors in explaining susceptibility to smoking. The variable with the largest effect was how often the respondent is exposed to smoking in the same room or car as the subject. Those with frequent exposure (at least weekly) were much more likely to be susceptible than those not exposed in the last year (odds ratio with 95% confidence interval (OR) 4.2[2.0,9.9]). Having at least one friend who is a smoker was also statistically significant (*p*<0.01) in predicting susceptibility. Neither living with a smoker or having at least one family member who is a smoker was statistically significant (*p*>0.05), adjusting for the other variables.

Model 3 jointly modelled rebelliousness, having a friend who is a smoker, and frequency of exposure to smoking with the two diffusion measures as predictors of susceptibility. While neither of the diffusion of susceptibility measures was statistically significant (*p*>0.05), model 3 was a better fit than model 2 (AIC improves from 429 to 383).

### Change in smoking susceptibility

Of 384 students for whom data were available, 78 changed their susceptibility status between pre-intervention and post-intervention (see Table 3). Similarly, 70 of 363 responding students changed their susceptibility status between post-intervention and follow-up. These status changes occurred in both directions, with a slight increase in the number susceptible over time in the control group, consistent with the vulnerability of this age group to becoming smokers.

**Table 3:** Susceptible proportion of nominated friends, by susceptibility pattern

Susceptible?	Intervention group		Control group	
	n	Susceptible	n	Susceptible
Pre-intervention to Post-intervention				
No to No	112	29%	103	25%
No to Yes	22	36%	22	33%
Yes to No	17	42%	17	36%
Yes to Yes	48	48%	43	49%
missing	44		52	
Post-intervention to Follow-up				
No to No	108	25%	93	26%
No to Yes	16	44%	23	45%
Yes to No	20	49%	11	40%
Yes to Yes	51	47%	41	45%
missing	48		69	

After adjusting for existing susceptibility and any effects of the intervention, there was no consistently significant relationship between future susceptibility and any of the modelled measures (see Table 4 for results). Two social environment measures that were associated with susceptibility at pre-intervention, were also found to be significantly associated with change of susceptibility: friends with a smoker for pre-intervention to post-intervention, and frequently in the presence of smoking for post-intervention to follow-up. Of particular interest for this study, changes in susceptibility were not found to be associated with the proportion of nominated friends who are susceptible to smoking, the indicator of hypothesised behaviour diffusion.

**Table 4:** Odds ratios for susceptibility to smoking by selected characteristics (with 95% confidence interval), adjusting for susceptibility at previous time point

	Pre-/post-intervention	Post-/follow-up
Existing susceptibility	10.7 (5.8-20.5)	8.6 (4.6-16.8)
Intervention: Control	Reference	Reference
Intervention: Intervention	0.8 (0.4-1.6)	0.7 (0.3-1.3)
Rebelliousness	1.1 (0.9-1.2)	1.1 (1.0-1.2)
Friends with a smoker	2.8 (1.4-5.3)	1.4 (0.7-2.7)
Smoking presence: Never	Reference	Reference
Smoking presence: Sometimes	1.0 (0.4-2.5)	1.9 (0.8-4.9)
Smoking presence: Frequently	1.2 (0.5-2.8)	3.0 (1.3-7.7)
Nominated friends susceptible	2.3 (0.5-9.0)	1.4 (0.4-5.6)
Nominators susceptible	0.7 (0.2-2.4)	3.5 (1.0-12.8)

^1^The relevant time points are pre-intervention (pre-), post-intervention (post-) and follow-up

We constructed stochastic actor-oriented models to more formally explore the potential role of the nominated friends network in diffusing susceptibility. Only 10 of the 20 classes (4 control and 6 intervention) provided data of sufficient completeness for modelling, with at least 80% of students nominating at least one friend in all three waves. However, model convergence was poor and the analysis provided no evidence about the potential role of susceptibility of friends in influencing smoking susceptibility through network diffusion. Full details are at Additional file 2.

## Discussion

This study highlights the important influence of an adolescent’s social environment on their attitudes and propensity to take up smoking. There are many features of the social environment that could affect the way in which such influence is realised. In this study, these features include:

- source of influence: family members, friends, and friends within the class;
- content: their smoking behaviour, and attitude as measured by the susceptibility construct; and
- transparency: whether the person being influenced can perceive the content.

The influence of friends and family as role models for smoking is already established [3, 5]. Behavioural interventions that are delivered to individuals are only able to address such influences indirectly. For example, one of the four Dead Cool video and discussion sessions focused on the effect of such influences [14], but there was no attempt to educate parents so as to reduce exposure of participants to their parents’ smoking.

School based interventions directed to individuals also affect the social environment of participants simply because some friends may also be participants. The way in which the school environment interacts with other elements of the social environment is poorly understood. This study adds to the evidence base for designing complex interventions that operate more broadly than the individual. The smoking status of friends and the frequency of being in the close presence of smoking were both found to be significantly associated with susceptibility and with change in susceptibility (adjusted for any effects of the Dead Cool intervention).

There is growing recognition of the need to consider behaviour in context [22], and in developing interventions that explicitly address the social environment. Network interventions [23] consider friendship or other social networks in the intervention design. Broader policy initiatives such as the October 2015 United Kingdom ban of smoking in cars where minors (aged to 18) are present may also impact on the social environment. While this was introduced to reduce passive smoking, compliance would also reduce the frequency of children and adolescents being in the presence of smoking. The analysis of the Dead Cool study suggests that individually delivered interventions would be most effective as part of a programme that also includes such broader initiatives.

With regard to the potential diffusion of susceptibility through social networks, this study does not provide any evidence to support such diffusion. This outcome should not be taken as evidence of no contagion as the methods we used for assessing the association between the susceptibility of individuals and their friends were relatively insensitive, given that too few students changed susceptibility over the observation period. That is, the results suggest that the dataset was underpowered for this particular research question, a common difficulty when fitting models with co-evolving networks and behaviour [24].

As Dead Cool was delivered at the school class level, friendship network data was only available for class, limiting the study. Students may have different classmates for different subjects, based on ability or subject choice. Thus, the class in which Dead Cool was delivered and therefore the network nominated may not identify the strongest peer influences. The significant association between susceptibility and the smoking status of unspecified friends supports the interpretation that there is limited overlap between the two types of friends in this study. Adolescent risk behaviours have been shown to be more strongly associated with friendship networks where the boundary is set more broadly [25], and future studies would benefit by including full school years instead of selected classes within a school year.

The limited power was exacerbated by the high level of missing data for susceptibility (see Tables 1 and 3), the attitude measure to be explained. Similar analysis on a larger purpose-specific dataset would be required that includes a range of attitude measures.

In combination, these results suggest that influential strength is affected by source, content, and transparency. In this study, modelling of smoking behaviour by friends and family is influential while there is no evidence that the more restricted group of friends within a classroom are able to influence attitudes as measured by susceptibility.

## Conclusions

This study provides further evidence that the social environment influences adolescents in their uptake of smoking, over and above the contribution of personal characteristics, with frequency of being in the presence of smoking (in the same room or car) a particularly important contributor. The analysis supports policy initiatives that alter the social environment, such as banning smoking in cars where children are present.

This study does not provide any evidence concerning diffusion of susceptibility through friendship networks in schools. This result suggests that mechanisms of influence that involve modelling of behaviour, such as regular observation of smoking, are stronger than potential mechanisms that involve people unconsciously adopting their friend’s attitudes, at least in the case of smoking uptake by adolescents. To properly assess the potential of susceptibility diffusion over social networks, larger studies are required that allow broader friendship nominations and, potentially, also assess attitudes at a younger age.

## Additional files

Additional file 1 Dead Cool Questionnaire. Survey instrument used to collect demographic, psychosocial, social network and smoking related behaviour data. (PDF 153 kb)

Additional file 2 The importance of social environment in preventing smoking: RSiena analysis. Analysis of the co-evolution of network and smoking susceptibility using Stochastic Actor Orientated Models. (PDF 108 kb)

## Data Availability

General data from the Dead Cool study are included in [14]. The network data using in this study are not included due to risk of identification.

## Abbreviations

AIC: Akaike information criterion
BIC: Bayesian information criterion
OR: Odds ratio

## References

[1] Conrad KM, Flay BR, Hill D (1992). Why children start smoking cigarettes: predictors of onset. Addiction.

[2] Wang MQ, Fitzhugh EC, Westerfield RC, Eddy JM (1995). Family and peer influences on smoking behavior among american adolescents: An age trend. J Adolesc Health.

[3] Leonardi-Bee J, Jere ML, Britton J (2011). Exposure to parental and sibling smoking and the risk of smoking uptake in childhood and adolescence: a systematic review and meta-analysis. Thorax.

[4] Hoffman BR, Sussman S, Unger JB, Valente TW (2006). Peer influences on adolescent cigarette smoking: A theoretical review of the literature. Subst Use Misuse.

[5] Vitória PD, Salgueiro MF, Silva SA, de Vries H (2011). Social influence, intention to smoke, and adolescent smoking behaviour longitudinal relations. Br J Health Psychol.

[6] Bandura A (1986). Social Foundations of Thought and Action: A Social Cognitive Theory.

[7] Seo D-C, Huang Y (2011). Systematic review of social network analysis in adolescent cigarette smoking behavior. J Sch Health.

[8] Liu J, Zhao S, Chen X, Falk E, Albarracín D (2017). The influence of peer behavior as a function of social and cultural closeness: A meta-analysis of normative influence on adolescent smoking initiation and continuation. Pol Psychol Bull.

[9] Mercken L, Snijders TAB, Steglich C, de Vries H (2009). Dynamics of adolescent friendship networks and smoking behavior: Social network analyses in six european countries. Soc Sci Med.

[10] Valente TW, Carrington PJ, Scott J, Wasserman S (2005). Network models and methods for studying the diffusion of innovations. Models and Methods in Social Network Analysis.

[11] Fishbein M (1995). Developing effective behavior change interventions: some lessons learned from behavioral research. NIDA Res Monogr.

[12] Foster C, Scarlett M, Stewart B. Young persons’ behaviour and attitude survey 2016: Health modules. Tech Rep Dep Health. 2017. https://www.health-ni.gov.uk/sites/default/files/publications/health/bulletin-16-ypbas.pdf. Accessed 21 Sept 2017.

[13] NISRA: Central Survey Unit. Young persons’ behaviour & attitudes survey 2016 topline results (weighted). Tech Rep North Irel Stat Res Agency. 2017. https://www.nisra.gov.uk/sites/nisra.gov.uk/files/publications/YPBAS2016ToplineResults.pdf. Accessed 21 Sept 2017.

[14] Thurston A, Dunne L, Kee F, Gildea A, Craig N, Stark P, Lazenbatt A. (2019). A randomized controlled efficacy trial of a smoking prevention programme with grade 8 students in high schools. Int J Educ Res.

[15] Pierce JP, Choi WS, Gilpin EA, Farkas AJ, Merritt RK (1996). Validation of susceptibility as a predictor of which adolescents take up smoking in the United States. Health Psychol.

[16] R Core Team (2015). R: A Language and Environment for Statistical Computing.

[17] Handbook of Adolescent Health Risk Behavior In: DiClemente RJ, Hansen WB, Ponton LE, editors. New York: Springer: 2013.

[18] NISRA. Northern Ireland Multiple Deprivation Measure 2010 (statistical geographies). North Irel Stat Res Agency. 2010. Downloaded on 21 September 2017.

[19] Snijders TA, Steglich C, Schweinberger M, van Montfort K, Oud H, Satorra A (2007). Modeling the Coevolution of Networks and Behavior. Longitudinal Models in the Behavioral and Related Sciences.

[20] Steglich C, Snijders TAB, Pearson M (2010). Dynamic Networks and Behavior: Separating Selection from Influence. Sociol Methodol.

[21] Ripley RM, Snijders TAB, Zsófia Boda AV, Preciado P. Manual for SIENA Version 4.0. University of Oxford, Department of Statistics; Nuffield College. 2018. http://www.stats.ox.ac.uk/~snijders/siena.

[22] Berkman LF, Glass T, Brissette I, Seeman TE (2000). From social integration to health: Durkheim in the new millennium. Soc Sci Med.

[23] Valente TW (2012). Network interventions. Science.

[24] Stadtfeld C, Snijders TAB, Steglich C, van Duijn M. Statistical power in longitudinal network studies. Sociol Methods Res. 2018. 10.1177/0049124118769113.

[25] Valente TW, Fujimoto K, Unger JB, Soto DW, Meeker D (2013). Variations in network boundary and type: A study of adolescent peer influences. Soc Networks.

