# Supplementary material for: The importance of social environment in preventing smoking: an analysis of the Dead Cool intervention
Source: BMC Public Health. 2019 Aug 28;19:1182. doi: 10.1186/s12889-019-7485-7 (PMC6714405; doi:10.1186/s12889-019-7485-7)
Supplement: Supplementary file 1 — Dead Cool Questionnaire. Survey instrument used to collect demographic, psychosocial, social network and smoking related behaviour data. (PDF 153 kb) [file 12889_2019_7485_MOESM1_ESM.pdf]

22. Please say how much you agree or disagree with the following sentences *(Please circle your answer)*

My life is going well

Strongly agree | Tend to agree | Neither agree nor disagree | Tend to disagree | Strongly disagree | Don't know

My life is just right

Strongly agree | Tend to agree | Neither agree nor disagree | Tend to disagree | Strongly disagree | Don't know

I wish I had a different kind of life

Strongly agree | Tend to agree | Neither agree nor disagree | Tend to disagree | Strongly disagree | Don't know

I have a good life

Strongly agree | Tend to agree | Neither agree nor disagree | Tend to disagree | Strongly disagree | Don't know

I have what I want in life

Strongly agree | Tend to agree | Neither agree nor disagree | Tend to disagree | Strongly disagree | Don't know

23. How much do the following statements describe you? *(Please circle your answer)*

I get in trouble in school

Exactly like me | Pretty much like | A little like me | Not at all like me

I do things my parents wouldn't want me to do

Exactly like me | Pretty much like | A little like me | Not at all like me

I like scary things

Exactly like me | Pretty much like | A little like me | Not at all like me

I like to do dangerous things

Exactly like me | Pretty much like | A little like me | Not at all like me

24. Have you ever stayed away from school without permission (truanted/bunked off)?

☐ Yes ☐ No ☐ Don't know

25. Do you think school has given you enough information on smoking?

☐ Yes ☐ No ☐ Don't know

26. How should the school offer advice and support on smoking?

____

27. Do you get regular pocket money (from parents, relatives?)

☐ Yes ☐ No

28. Can you spend your money however you like?

☐ Yes ☐ No

www.qub.ac.uk/edu

Queen's University Belfast

SCHOOL OF Education

DEAD COOL SURVEY

SCHOOL: ______________________________

STUDENT'S FULL NAME: ______________________________

DATE OF BIRTH: ______________________________

OFFICE USE ONLY

SCHOOL CODE: ______________________________

PUPIL CODE: ______________________________

Thanks you for agreeing to take part in our study. We promise that your answers are confidential. They will not be shown to anyone that you know.

Example questions:
Most of the questions can be answered by putting a tick in the box next to the answer that applies to you. Sometimes you can circle the answer. Or sometimes you have to write a number in the box. Sometimes you have to write in an answer, for example: What is your favourite colour? Blue

1. Are you a boy or a girl?

____

2. What age are you?

____

3. What is your postcode e.g. BT12 8NF?

____

4. What is your ethnic group?
Tick one box only, the one that is most true for you

☐ White British
☐ Asian
☐ African
☐ Chinese
☐ White Irish
☐ Any other ethnic group (please write in)

____

5. Who do you live with? *(please tick all that apply)*

☐ Mother
☐ Sisters/brothers
☐ Father
☐ Foster care
☐ Step-parent
☐ (Other please write in)

____

www.qub.ac.uk/edu

Edu_DeadCoolSurvey.indd 1-2 18/03/2015 10:28

The next questions are about cigarettes. Remember that your name is not on the questionnaire, so no-one who knows you will find out your answers.

6. **Do you smoke cigarettes at all nowadays?**
   - ☐ Yes ☐ No

7. **Now read the following statements carefully and tick the box next to the one which best describes you.**
   - ☐ I have never smoked
   - ☐ I have only ever tried smoking once
   - ☐ I used to smoke sometimes but I never smoke a cigarette now
   - ☐ I sometimes smoke cigarettes now but I don't smoke as many as one a week

7. **Just to check, read the statements below carefully and tick the box next to the one which best describes you.**
   - ☐ I have never tried smoking a cigarette, not even a puff or two
   - ☐ I did once have a puff or two of a cigarette, but I never smoke now
   - ☐ I do sometimes smoke cigarettes

8. **If you DO smoke, please answer the next question.** *If not, please skip to Question 10.*
   - ☐ I usually smoke between one and six cigarettes a week
   - ☐ I usually smoke more than six cigarettes a week
   - ☐ How many cigarettes do you smoke in a week?

9. **How old were you when you first tried smoking a cigarette, even if it was only a puff or two? Write your age in the box.** *If you have never smoked, please skip to Question 10.*

   I was________ years old.

10. **How do you think your family would feel if you started smoking?** *Tick all that apply.*
    - ☐ They would try to stop me
    - ☐ They would try to persuade me not to smoke
    - ☐ They would do nothing
    - ☐ They would encourage me to smoke
    - ☐ I don't know

11. **In the past year, have you seen cigarette packets on display in any of the shops listed below?** *Tick all that apply.*
    - ☐ A supermarket
    - ☐ A newsagent, tobacconist or a sweet shop
    - ☐ A petrol station or garage shop
    - ☐ Some other type of shop
    - ☐ Have not seen cigarettes for sale in any of these places

12. **Do any of these people that you know smoke cigarettes?** *Tick all that apply*
    - ☐ My boyfriend or girlfriend
    - ☐ Some friends of my own age
    - ☐ Some friends older than me
    - ☐ Some friends younger than me
    - ☐ My mother
    - ☐ My father
    - ☐ My step-parent
    - ☐ My brother or sister
    - ☐ Other relatives
    - ☐ None of my friends or family smoke

13. **Can you name your five closest friends in your school class?** *Please put a mark beside your best friend's name.*

    1. ____________________
    2. ____________________
    3. ____________________
    4. ____________________
    5. ____________________

14. **In the past year, how often were you in the same room as someone smoking at home?**
    - ☐ Every day or most days
    - ☐ Once or twice a week
    - ☐ Once or twice a month
    - ☐ Less often than once a month
    - ☐ Never in the past year
    - ☐ Don't know

15. **In the past year, how often were you in the same room as someone smoking at someone else's home?**
    - ☐ Every day or most days
    - ☐ Once or twice a week
    - ☐ Once or twice a month
    - ☐ Less often than once a month
    - ☐ Never in the past year
    - ☐ Don't know

16. **In the past year, how often were you in your family's car with somebody smoking?**
    - ☐ Every day or most days
    - ☐ Once or twice a week
    - ☐ Once or twice a month
    - ☐ Less often than once a month
    - ☐ Never in the past year
    - ☐ Don't know

17. **In the past year, how often were you in someone else's car with somebody who was smoking?**
    - ☐ Every day or most days
    - ☐ Once or twice a week
    - ☐ Once or twice a month
    - ☐ Less often than once a month
    - ☐ Never in the past year
    - ☐ Don't know

18. **Do you think you will try a cigarette soon?**
    - ☐ Yes ☐ No ☐ Don't know

19. **If one of your best friends were to offer you a cigarette, would you smoke it?** *(Please tick your answer)*
    - ☐ Definitely yes ☐ Probably not
    - ☐ Probably yes ☐ Definitely not
    - ☐ Not sure

20. **Do you think you will smoke a cigarette at any time in the next year?** *(Please tick your answer)*
    - ☐ Definitely yes ☐ Probably not
    - ☐ Probably yes ☐ Definitely not
    - ☐ Not sure

21. **Against each statement, circle either TRUE or FALSE**

People of my age smoke because it helps them to relax	**TRUE**	**FALSE**
People of my age smoke because they are addicted to cigarettes	**TRUE**	**FALSE**
People of my age smoke because they believe it helps them to stay slim	**TRUE**	**FALSE**
People of my age smoke because it helps them to cope with stress in their life	**TRUE**	**FALSE**
People of my age smoke to look cool in front of their friends	**TRUE**	**FALSE**
People of my age smoke because they find it exciting to break the rules	**TRUE**	**FALSE**
People of my age smoke because their friends pressure them into it	**TRUE**	**FALSE**

Edu_DeadCoolSurvey.indd 3-4 18/03/2015 10:28
