# Supplementary material for: The importance of social environment in preventing smoking: an analysis of the Dead Cool intervention
Source: BMC Public Health. 2019 Aug 28;19:1182. doi: 10.1186/s12889-019-7485-7 (PMC6714405; doi:10.1186/s12889-019-7485-7)
Supplement: Supplementary file 2 — The importance of social environment in preventing smoking: RSiena analysis. Analysis of the co-evolution of network and smoking susceptibility using Stochastic Actor Orientated Models. (PDF 108 kb) [file 12889_2019_7485_MOESM2_ESM.pdf]

The importance of social environment in preventing smoking: RSiena analysis

Methods

Stochastic Actor Orientated Models (SAOMs) are a methodology which allows for the co-evolution of both network and behavioural dynamics, which account for the processes of selection and influence in the evolution of the friendship network and behaviour of the actors. It is processed within RSiena (Simulations Investigation for Empirical Network Analysis), which provides estimates for the dynamics while accounting for endogeneity between these features (1).

We included three standard network structure two application specific attributes in our model of network formation (see Network Dynamics variables in Tables 1, 2 and 3). The standard explanatory processes are: density - the tendency for friendship to be selective or precious; reciprocity - for friendship to be mutual/two-way; transitivity- a friend of a friend to be your friend (via transitive triplets and 3-cycles). The attribute based formation variables represent the effect of two people selecting each other as friends on the basis of a similarity: we included rebellion similarity and gender similarity.

The model also includes changes in the behaviour of interest, susceptibility (see Behaviour Dynamics variables in Tables 1, 2 and 3). One term allows for a linear trend over time (linear outcome). Two behaviour effects model the influence of the friendship network on susceptibility. The 'average alter' effect is the preference to be similar in behaviour to the average behaviour of one's nominated friends, in other words the influence process. The 'indegree' is any influence arising from the number of nominations received, that is the influence of popularity. Positive estimates would imply such tendencies.

The standard approach is to run the same model on each class network, as they are independent. Network data should be 80% complete at all waves to ensure suitable stability is reached, and three runs completed to improve convergence (output from run one is input to run two etc, as this is a stochastic approach). Estimates and SE for each network and behaviour element are given, with a convergence ratio < 0.1 ideal, but < 0.2 is considered moderate and accepted.

After individual SOAMs are run, a meta analysis across the various networks can be carried out to view the entire processes occurring and investigate if heterogeneity exists across the data.

Table 1: RSiena model results for class 10C

Effect	par.	(s.e.)
Network Dynamics		
constant friendship rate (period 1)	8.403	(1.380)
constant friendship rate (period 2)	7.112	(1.354)
outdegree (density)	-1.732	(0.132)
reciprocity	0.926	(0.226)
transitive triplets	0.240	(0.054)
3-cycles	-0.171	(0.118)
rebellion similarity	0.171	(0.298)
gender similarity	1.009	(0.215)
Behaviour Dynamics		
rate susceptibility (period 1)	0.974	(0.652)
rate susceptibility (period 2)	0.286	(0.207)
susceptibility linear shape	-0.169	(1.954)
susceptibility indegree	-0.211	(0.451)
susceptibility average alter	3.507	(5.795)

convergence t ratios all < 0.07.

Overall maximum convergence ratio 0.2.

Results

For each of the 10 suitable classes, we ran the model three times, with the second and third runs initialised at the solution of the prior run. In the event, model convergence was poor. While the specific outcome is stochastic, an example set of models found: 3 models converged, 4 models converged but with unacceptable fit statistics, 2 models failed to converge, and 1 model had such poor convergence that the algorithm failed to complete. The number of susceptibility changes in each class ranged between 3 and 14 (with each student potentially having two). The models that converged were concentrated in the classes with the higher number of changes, suggesting that the study was underpowered for this analysis.

The results from the converging models for one set of runs are displayed.

References

[1] Steglich, C., Snijders, T.A.B., Pearson, M.: Dynamic Networks and Behavior: Separating Selection from Influence. Sociological Methodology **40**(1), 329–393 (2010). doi:10.1111/j.1467-9531.2010.01225.x

Table 2: RSiena model results for class 13C

Effect	par.	(s.e.)
Network Dynamics		
constant friendship rate (period 1)	3.871	(0.630)
constant friendship rate (period 2)	5.663	(0.849)
outdegree (density)	-2.053	(0.165)
reciprocity	1.545	(0.260)
transitive triplets	0.362	(0.075)
3-cycles	-0.282	(0.139)
rebellion similarity	0.232	(0.378)
gender similarity	0.677	(0.262)
Behaviour Dynamics		
rate susceptibility (period 1)	1.580	(1.112)
rate susceptibility (period 2)	1.404	(1.138)
susceptibility linear shape	0.752	(1.505)
susceptibility indegree	-0.439	(0.401)
susceptibility average alter	0.554	(3.321)

convergence t ratios all < 0.11.

Overall maximum convergence ratio 0.23.

Table 3: RSiena model results for class 15C

Effect	par.	(s.e.)
Network Dynamics		
constant friendship rate (period 1)	4.266	(0.654)
constant friendship rate (period 2)	4.835	(0.714)
outdegree (density)	-1.888	(0.154)
reciprocity	0.976	(0.216)
transitive triplets	0.232	(0.061)
3-cycles	-0.126	(0.110)
rebellion similarity	0.182	(0.359)
gender similarity	1.317	(0.281)
Behaviour Dynamics		
rate susceptibility (period 1)	1.241	(0.928)
rate susceptibility (period 2)	0.754	(0.492)
susceptibility linear shape	-1.278	(2.095)
susceptibility indegree	-0.049	(0.402)
susceptibility average alter	9.366	(13.411)

convergence t ratios all < 0.09.

Overall maximum convergence ratio 0.23.
